# Dr. Henry Joslyn

**Published:** 1899-03

**Authors:** 


Dr. Henry Joslyn, U. of B. '98, died at Denver, Col., November 27, 1898, of septic endocarditis. After graduation he went to Denver, where he located and soon afterward was appointed instructor in dermatology in the medical department of the University of Denver.

Dr. Joslyn was born at Winchester, Eng., in 1862. He obtained his preliminary education at Winchester's famous school and college, and soon thereafter came to America. His medical training was received in the medical department of the University of Buffalo, at Dublin's celebrated Rotunda Hospital, and in London clinics. During the latter period of his medical instruction, he paid especial attention to diseases of the skin. He was a young man of great promise and his early death is deeply lamented by his colleagues and many friends.

